# Chronic Somatic Comorbidity and Excess Mortality Due to Natural Causes in Persons with Schizophrenia or Bipolar Affective Disorder

**DOI:** 10.1371/journal.pone.0024597

**Published:** 2011-09-14

**Authors:** Thomas Munk Laursen, Trine Munk-Olsen, Christiane Gasse

**Affiliations:** National Centre for Register-Based Research, University of Aarhus, Aarhus, Denmark; University of Adelaide, Australia

## Abstract

**Background:**

Suicide and death by accidents in persons with schizophrenia and bipolar disorder are common, but excess mortality from natural death accounts for even more years of life lost. The impact of somatic comorbidity, however, often is not duly considered in analyses and explanations of excess mortality in patients with psychotic disorders.

**Objective/Methods:**

This study investigates and evaluates the impact of 19 severe chronic diseases on excess mortality due to diseases and medical conditions (natural death) in individuals with psychotic disorders compared with the general population using a population-based cohort study in Denmark. Incidence/mortality rate ratios of admission/mortality were calculated using survival analysis.

**Results:**

Cohort members with psychotic disorders had higher incidence rates of hospital contacts for almost all of the 19 disorders than the general population. The mortality rate ratio (MRR) of natural death was 7.10 (95% CI 6.45, 7.81) for schizophrenic men, decreasing to 4.64 (95% CI 4.21, 5.10) after adjustment for the somatic disorders. The same pattern existed in women and in both genders with bipolar disorder. Highest MRRs were observed for psychotic patients without hospital admissions with the investigated somatic disorders.

**Conclusion:**

Chronic somatic diseases accounted for half of the excess mortality in patients with schizophrenia or bipolar disorder. Chronic disorders investigated in this paper seem to be under-treated or under-detected among such patients.

## Introduction

Premature death in persons with schizophrenia or bipolar disorder is well documented, both in descriptions dating back in history [1] and in recent studies [2]–[6]. Excess mortality from diseases and medical conditions (death from natural causes) accounts for even more years of life lost in these patients than do suicide and death by accidents [7]. Side-effects of pharmacological treatment [8], unhealthy diet [9], and high levels of cigarette smoking [10], [11], as well as inadequate medical treatment or provision of health care [12], [13] have been proposed as probable reasons for the excess mortality from natural causes among psychotic patients. In particular, unfavourable life style factors as well as antipsychotic treatment are associated with a range of somatic disorders, such as diabetes, chronic obstructive pulmonary disease, and cardiovascular disease, which may constitute the immediate cause of premature death from natural causes. However, the impact of somatic comorbidity is often not duly considered in analyses and explanations of the excess mortality [14].

To contribute to the discussion about the causes of excess mortality among patients with schizophrenia and bipolar disorder, we investigated the incidence rates of 19 severe somatic chronic diseases among persons with psychotic disorders (schizophrenia or bipolar disorder), and compared these rates (incidence rate ratios) with those of persons with no record of psychiatric hospital admission or outpatient contact. Furthermore, we evaluated the impact of these conditions on the estimates of the excess mortality (mortality rate ratios) from natural causes.

## Methods

### Data source and study population

We conducted a population-based cohort study using Danish register data. The study population comprised all persons born in Denmark from 1 January 1955 to 1 June 1992 (thus at least 15 years old in 2007) and residing in Denmark at some point during the period 1995 to 2006. They were identified from The Danish Civil Registration System [15]. The Civil Registration System was established in 1968, at which time all persons living in Denmark were registered and assigned a 10-digit personal identification number enabling data-linkage. The register records information on gender, date of birth, place of birth, and vital status (e.g., date of death and migration out of Denmark).

Data on psychiatric hospitalizations of the entire Danish population were drawn from the Danish Central Psychiatric Case Register [16], which is computerized since 1969, and were linked with the study population using the personal identification number. Patients with schizophrenia (ICD8: 295 (exclusive 295.79), ICD10: F20) and bipolar disorder (ICD8: 296.39, 296.19, ICD10: F30, F31) and with any of the remaining psychiatric diagnoses registered at psychiatric contact were indentified. Persons were categorized as having schizophrenia/bipolar disorder or any other psychiatric diagnosis from the date of their first contact (i.e. out- or inpatient contact) to a psychiatric hospital with such a diagnosis. Thus, individuals with a psychiatric contact before 1995, e.g. due to schizophrenia, would be in the group of persons with schizophrenia throughout the follow-up period. All Danish citizens have access to public health care free of charge. Severe mental disorders are only treated in public hospitals. This ensures almost total coverage of contacts with psychiatric disorder in the registers.

We applied the Charlson Comorbidity Index [17], [18] for assessing severe chronic somatic diseases. The Index includes 19 severe, chronic diseases to which a weight from one to six to each disorder is assigned according to the severity of the disease (Table 1). The score of the Index is the sum of all weights. The Index was originally constructed to measure the impact of comorbidity on mortality in a hospital setting among breast cancer patients [17] and it has later been adapted to fit with ICD-10 diagnoses [18].

**Table 1:** Number of cases and incidence rates per 1000 person-years under risk for four subgroups of the cohort of the 19 chronic diseases included in the Charlson Comorbidity Index [18].

Chronic somatic disorder	Weight	Schizophrenia	Bipolar disorder	Other psychiatric diseases	No psychiatric admissions
		N (cases/1000 PY)	N (cases/1000 PY)	N (cases/1000 PY)	N (cases/1000 PY)
Myocardial infarction	1	74 (0.58)	23 (0.57)	799 (0.63)	6156 (0.25)
Congestive heart failure	1	91 (0.72)	28 (0.69)	493 (0.39)	3065(0.13)
Peripheral vascular disease	1	66 (0.52)	18 (0.45)	950 (0.75)	7443 (0.31)
Cerebrovascular disease	1	167 (1.32)	77 (1.93)	2202 (1.74)	14501 (0.60)
Dementia	1	19 (0.15)	14 (0.35)	228 (0.18)	436 (0.02)
Chronic pulmonary disease	1	481 (3.95)	126 (3.24)	4560 (3.76)	38653 (1.63)
Connective tissue disease	1	73 (0.58)	21 (0.52)	1570 (1.25)	18154 (0.75)
Ulcer disease	1	232 (1.85)	62 (1.55)	2565 (2.05)	13458 (0.56)
Mild liver disease	1	318 (2.54)	82 (2.05)	3330 (2.67)	6647 (0.27)
Diabetes I and II	2	372 (2.98)	74 (1.86)	1711 (1.36)	12950 (0.54)
Hemiplegia	2	26 (0.20)	15 (0.37)	340 (0.27)	2054 (0.08)
Moderate to severe renal disease	2	108 (0.85)	44 (1.10)	794 (0.63)	6085 (0.25)
Diabetes with end organ damage	2	135 (1.07)	23 (0.57)	900 (0.71)	6258 (0.26)
Any tumor	2	214 (1.70)	85 (2.13)	2024 (1.60)	26048 (1.08)
Leukemia	2	10 (0.08)	2 (0.05)	63 (0.05)	885 (0.04)
Lymphoma	2	18 (0.14)	5 (0.12)	182 (0.14)	2195 (0.09)
Moderate to severe liver disease	3	57 (0.45)	18 (0.45)	961 (0.76)	1582 (0.07)
Metastatic solid tumor	3	40 (0.31)	10 (0.25)	442 (0.35)	4798 (0.20)
AIDS	6	33 (0.26)	3 (0.07)	245 (0.19)	901 (0.04)

Data on hospital contacts with the chronic somatic diseases included in the Charlson Comorbidity Index (Table 1) of the entire Danish population were drawn from the Danish National Hospital Register [19] and linked to the study population by the personal registration number. The Danish National Hospital Register was established in 1977 and contains information on all Danish somatic inpatient hospital contacts.

The Charlson Comorbidity Index score was calculated by adding the individual weights of the particular diseases. The score was calculated in a time-dependent manner where inpatient contacts from 1977 onwards were used. From 1995 onwards, outpatient contacts were also included. Underlying causes of natural death were identified from the Causes of Death Register [20]. Psychiatric and somatic diseases were diagnosed and causes of death were determined using the ICD8 [21] classification system until 31 December 1993, and the ICD10 [22] classification from 1 January 1994. The ICD 9 classification was not used in Denmark.

### Statistical analyses

Outcomes of interest were the 19 chronic somatic diseases and death from natural causes. The follow-up began on 1 January 1995 or on the cohort member's 15th birthday, whichever came last. The follow-up ended on 1 July 2007, the day of death, the day of first admission with a chronic somatic disease (when this was the outcome), or the day of emigration, whichever came first.

In the calculations of IRRs of the 19 diseases of the Charlson Index, all persons admitted with the respective disorder from 1977 to 1994 were censored, while persons admitted with one of the disorders during follow-up were censored on the same day. This means that we only examined incident admissions for the respective diseases from 1995 on.

We calculated incidence rates and incidence rate ratios (IRRs) for the 19 somatic diseases and calculated mortality rate ratios (MRR) for natural death, adjusted or stratified by the Charlson Comorbidity Index score (in 5 categories: 0, 1, 2, 3, 4+), comparing persons with schizophrenia or bipolar disorder with persons without psychiatric hospital contacts. Poisson regression with the GENMOD procedure in SAS version 9.2 (SAS Institute Inc, Cary, NC) was used in the calculation of both IRR and MRR. This method approximates a Cox regression [23], [24].

All IRRs and MRRs were adjusted for or stratified by gender, calendar time, and age (adjustment for age was made in 5-year groups). IRRs and MRRs were calculated by log-likelihood estimation, and Wald's 95% confidence intervals were used.

The Charlson Comorbidity Index score was examined in the two oldest 5-year cohorts of the study population; those born in 1955–59 and those born in 1960–64. We calculated 95% confidence intervals (CIs) on the average Charlson score assuming a normal distribution.

## Results

In total, the study population comprised 2,450,812 persons between 15 and 52 years old at risk for admission with one of the 19 investigated somatic diseases and at risk for natural death. A total of 16,079 cohortees had been in contact with a psychiatric hospital with schizophrenia and 6,215 with bipolar affective disorder.

### Incidence rates and rate ratios of somatic diseases

**Figure 1:**
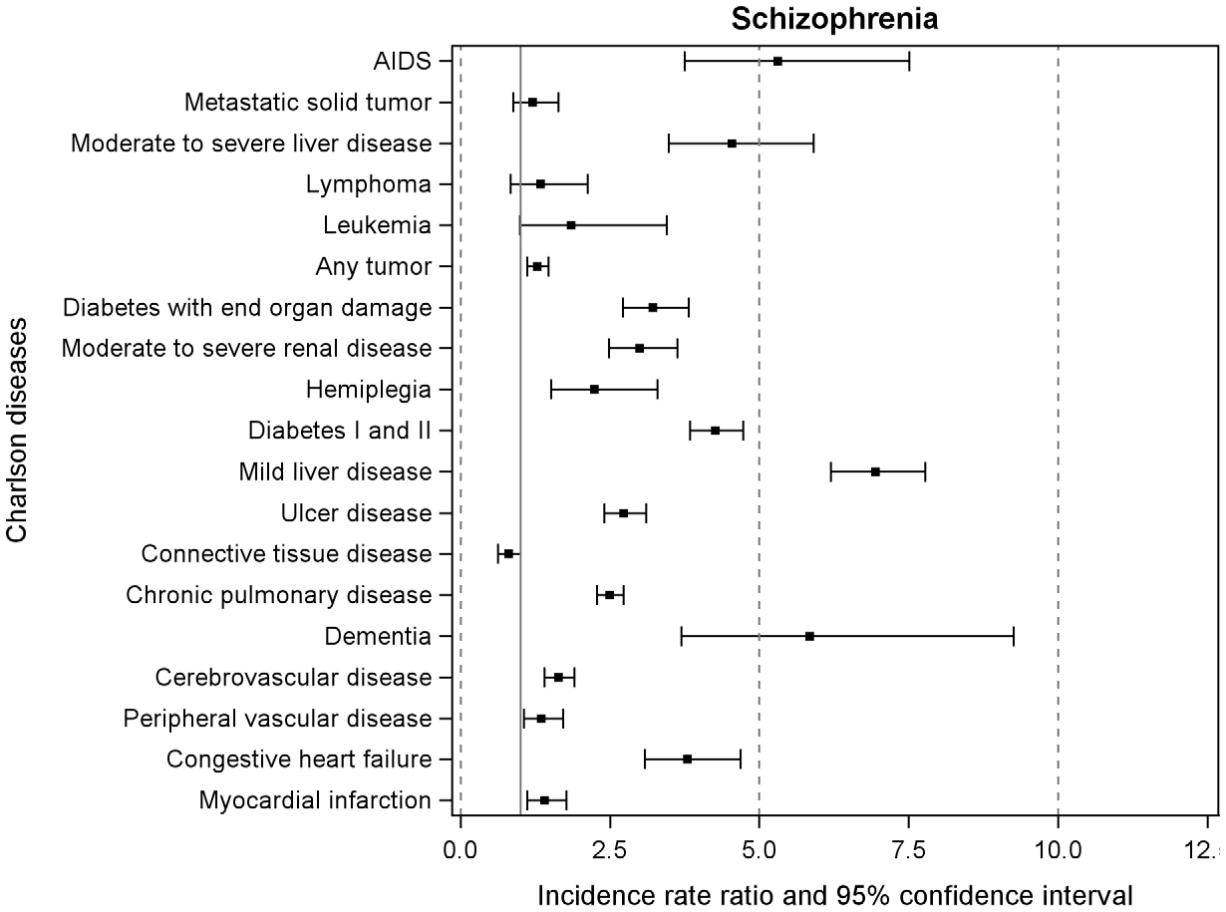
Incidence rate ratios of 19 somatic chronic diseases among patients with schizophrenia. Follow-up 1995–2007. Solid vertical line crossing at 1.0 is the reference including all persons never admitted to psychiatric hospital.

**Figure 2:**
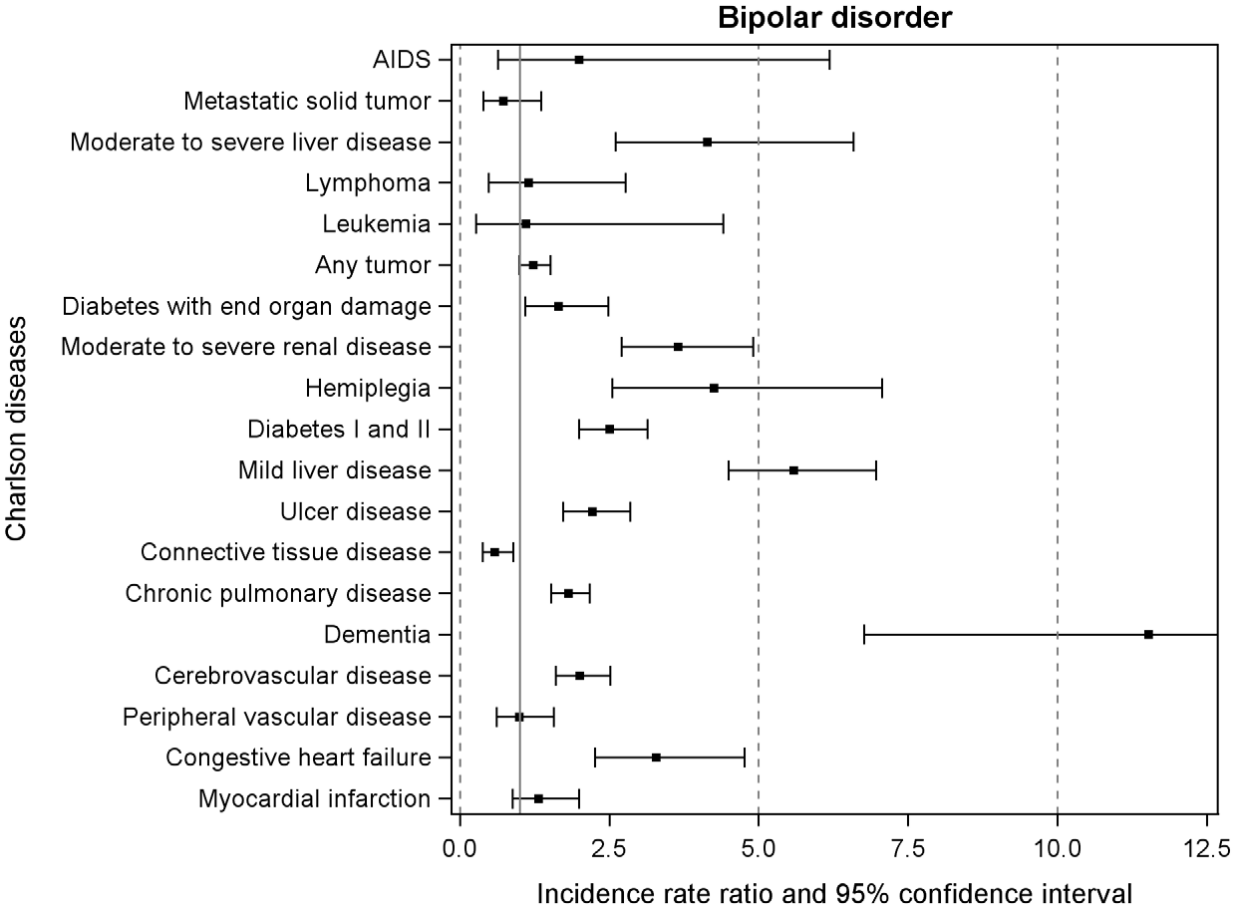
Incidence rate ratios of the 19 chronic somatic diseases among patients with bipolar disorder. Follow-up 1995–2007. Solid vertical line crossing at 1.0 is the reference including all persons never admitted to psychiatric hospital.

During the follow-up period from 1995 to 2007, the incidence rates (new cases per 1000 person years) of the individual 19 chronic somatic diseases were higher among persons with a psychiatric admission than among persons without psychiatric hospital admissions (Table 1). The IRRs of hospital contacts of all 19 somatic chronic disorders are displayed in Figures 1 and 2. The rates of all somatic disorders were higher (resulting in IRRs>1) among cohort members with schizophrenia or bipolar disorder than among individuals without psychiatric contacts, except for connective tissue disease in both disorders (IRR 0.80 (95% CI: 0.63, 1.00) and 0.58 (95% CI: 0.38, 0.89), respectively) and metastatic solid tumor in bipolar disorder (IRR 0.73 (95% CI: 0.39, 1.36)). Dementia and mild/severe liver disease had the highest IRR in both disorders: 5.84 (95% CI: 3.69, 9.25); 6.94 (95% CI: 6.20, 7.77), respectively in individuals with schizophrenia and 11.52 (95% CI: 6.76, 19.65); 5.59 (95% CI: 4.50, 6.96), respectively in individuals with bipolar disorder (Figures 1 and 2).

### Mortality rate ratios of natural death

The MRRs from natural causes were examined in two models with and without adjustment/stratification for the Charlson Comorbidity Index score in order to investigate the impact of the somatic diseases on the excess mortality. We found an MRR of 7.10 for men and 6.06 for women with schizophrenia without comorbidity adjustment. The MRRs were almost halved in the adjusted model, to 4.64 in men and 3.03 in women. The same pattern was present in bipolar disorder where the MRR dropped from 5.61 to 3.73 in men and from 3.71 to 2.44 in women, Table 2.

**Table 2:** Mortality rate ratio of death by natural causes in patients with previous hospital admissions/contact due to schizophrenia or bipolar disorder.

			Schizo-phrenia		Bipolar disorder		Never in contact*
		N	MRR	N	MRR	N	MRR
Men	Not adjusted for Charlson I.	451	7.10 (6.45, 7.81)	91	5.61 (4.56, 6.90)	7377	1 ref
	Adjusted for Charlson I.	451	4.64 (4.21, 5.10)	91	3.73 (3.03, 4.59)	7377	1 ref
Women	Not adjusted for Charlson I.	163	6.06 (5.19, 7.09)	59	3.70 (2.87, 4.80)	5170	1 ref
	Adjusted for Charlson I.	163	3.03 (2.60, 3.55)	59	2.44 (1.89, 3.15)	5170	1 ref

*: Comparison with cohortees who have never been in contact with a psychiatric hospital as reference (1 ref = 1 reference category). Follow-up 1995–2007.

Table 3 displays MRRs stratified by Charlson Comorbidity Index score categories. Among cohort members with a score equaling zero, indicating no recorded (treated) chronic somatic disorders, schizophrenic patients had an MRR of 12.71 compared with cohort members with no contact to a psychiatric hospital. Among cohort members with a score equaling one, the MRR was 4.98, declining to 2.59, 1.92, and 1.45, among cohort members with an index of 2, 3 and 4 or more, respectively. The same pattern was present among bipolar patients, Table 3.

**Table 3:** Mortality rate ratio of death by natural causes in patients with previous hospital admissions/contact due to schizophrenia or bipolar disorder.

		Schizophrenia		Bipolar disorder		Never in contact*
	N	MRR	N	MRR	N	MRR
Charlson index = 0	310	12.71(11.31, 14.28)	64	8.68 (6.78, 11.12)	3616	1 ref
Charlson index = 1	96	4.98 (4.05, 6.12)	28	4.23 (2.91, 6.16)	1524	1 ref
Charlson index = 2	71	2.59 (2.05, 3.28)	22	2.28 (1.50, 3.47)	2557	1 ref
Charlson index = 3	30	1.92 (1.33, 2.76)	6	1.18 (0.53, 2.64)	761	1 ref
Charlson index = 4+	107	1.45 (1.20, 1.76)	30	1.37 (0.96, 1.97)	4089	1 ref

*: Comparison with cohortees who have never been in contact with a psychiatric hospital as reference (1 ref = 1 reference category). Follow-up 1995–2007.

Stratified by Charlson index score.

### Somatic comorbidity score

We calculated the average Charlson Comorbidity Index score as of 1 July 2007 in the two oldest 5-year cohorts of the study population, i.e., those born 1955–59 and 1960–64, Table 4. In both sub-cohorts, cohort members with schizophrenia and bipolar disorder had an average score approximately twice that of the cohortees with no contact to a psychiatric hospital. Male cohortees with schizophrenia and bipolar disorder had a lower score than males with other psychiatric contacts.

**Table 4:** Average Charlson Index scores among men and women by birth cohort*.

		1955–1959 (age 48–52)	1960–1964 (age 43–47)
Men	Schizophrenia	0.61 (0.55–0.67)	0.46 (0.41–0.52)
	Bipolar disorder	0.60 (0.50–0.69)	0.33 (0.25–0.40)
	Other psych. adm.	0.77 (0.75–0.80)	0.54 (0.52–0.57)
	No psych. adm.	0.30 (0.30–0.31)	0.21 (0.21–0.22)
Women	Schizophrenia	0.64 (0.57–0.72)	0.47 (0.41–0.54)
	Bipolar disorder	0.55 (0.48–0.62)	0.36 (0.29–0.42)
	Other psych. adm.	0.64 (0.62–0.64)	0.47 (0.45–0.49)
	No psych. adm.	0.32 (0.31–0.32)	0.22 (0.22–0.22)

*: Score was calculated per 1 July 2007. Schizophrenia, bipolar disorder and other psychiatric disorders were measured as “lifetime” exposure (95% CI).

## Discussion

### Key findings

Cohort members with schizophrenia and bipolar disorder had higher incidence rates of hospital contacts with almost all of the investigated 19 chronic somatic disorders and had Charlson Comorbidity Index scores twice as high as persons without a record of psychiatric admission or outpatient contact. Chronic somatic comorbidity accounted for approximately half of the excess mortality due to natural causes in cohort members with schizophrenia and bipolar disorder. Stratification by the Charlson Comorbidity Index scores revealed that the MRRs among cohortees with schizophrenia and bipolar disorder were up to 13-fold increased in the strata with a Charlson Comorbidity Index scores equaling zero compared with cohortees with no contact to a psychiatric hospital, while the excess mortality decreased with increasing somatic comorbidity.

### Severe chronic somatic comorbidity as evaluated by the Charlson Comorbidity Index Score

The impact of somatic comorbidity is often not duly considered in the analysis and explanation of excess mortality among psychiatric patients [14]. In this paper, we addressed this issue by investigating 19 chronic somatic diseases and applying the Charlson Comorbidity Index.

Almost all somatic chronic disorders investigated in this study were more frequent in psychotic patients than in the general population. The only somatic disorder with a lower admission rate in psychotic patients was connective tissue diseases, mainly rheumatoid arthritis (with symptoms of musculoskeletal pain). This low admission rate was also noted in a previous Danish study, which concluded that reporting and detection of musculoskeletal pain in patients with schizophrenia was at a lower level compared with the general population [25]. Another Danish study found that schizophrenic patients had higher admission rates of acute, painful somatic disorders than non-schizophrenic patients, while they had lower rates of more chronic, less acute somatic disorders [26].

In our study excess mortality decreased by almost 50% after adjustment for somatic comorbidity indicating that approximately half of the excess mortality in men and women with schizophrenia and bipolar disease is rooted in diseases included in the Charlson Comorbidity Index. Note that if all of the excess mortality in psychotic patients had been attributable to the excess level of somatic illnesses included in the Charlson Index, we would expect the MRR of natural death to attenuate to one after Index adjustment. This was not the case. One explanation could be that psychotic patients more frequently harbor somatic disorders not included in the Charlson Comorbidity Index than does the general population, e.g. acute infections [4]. However, infections account for only 0.83% of the causes of death registered in the general Danish population, thus may only explain a smaller proportion of the remaining excess risk [27]. Other disorders not included in the index which are associated with excess mortality are epilepsy [28], or drug and alcohol abuse [29]. Substance abuse, for example, has been registered in 32% of patients with schizophrenia treated with antipsychotics in Denmark [30]. Although we did not directly adjust for alcohol and substance abuse and moreover smoking in this study, we adjusted for clinical conditions related to these risk factors such as COPD, liver diseases and dementia, which may serve as proxy measures for these factors.

In our opinion, the most plausible explanation that somatic comorbidity adjustment only halved the excess mortality is an under-detection of the chronic somatic diseases among psychotic patients. As shown in both Danish [13] and other studies [31], psychotic patients appear to be under-diagnosed with somatic disorders compared with the general population. This notion is also supported by our finding that the MRR of natural mortality was almost 13-fold increased among schizophrenic patients and almost 9-fold in bipolar patients with an index equaling 0 compared with persons who had never been in contact with psychiatric hospitals. Among all cohortees with a Charlson Comorbidity Index score equaling zero, many schizophrenic or bipolar patients may have undetected somatic diseases, which implies that they are compared within the same strata with more healthy cohortees without somatic disorders explaining the very high MRRs observed in the present study. The high MRRs could not be entirely explained by schizophrenic or bipolar patients having higher rates of somatic diseases not included in the Charlson Index as this would have resulted in excess mortality being more equally distributed among the different strata of the Charlson Comorbidity Index score.

### Comparison of somatic comorbidity with other studies

Our findings corroborate findings from previous studies from Australia and Canada reporting higher levels of somatic admissions in individuals with schizophrenia than in the general population [32], [33]. In a Danish study, schizophrenic patients had higher admission rates of acute, painful somatic disorders than non-schizophrenic patients, while they had lower rates of more chronic, less acute somatic disorders [26]. Furthermore, a higher prevalence of chronic medical conditions among patients with psychotic disorders has been shown in the U.S. [34], [35]. Moreover, the results presented here suggest the presence of under-detection or under-treatment of somatic disorders in this group. We interpret the lower than expected rates of hospital contacts as evidence of lack of sufficient somatic treatment, which could shift the MMR of natural death towards the high observed levels in our analysis. On the basis of our data analysis, we cannot determine reasons for the suggested under-treatment. One possible explanation is that patients were not sufficiently examined for which reason the somatic disorder was not discovered. Another explanation could be that the somatic disorder was found, but the patient was not referred to relevant treatment. It could also be that psychotic patients are more likely to refuse treatment than the general population.

### Register based studies, pros and cons

The diagnostic information in the Psychiatric Central Register for schizophrenia and bipolar disorder is not as accurate as information obtained in more structured research settings. However, a range of studies have shown strong agreement between the psychiatric clinical diagnoses in the registers and research criteria diagnoses [36]–[38].

In Denmark close to 100% of all diagnoses of schizophrenia and bipolar disorder are registered in the Psychiatric Central Register because the GPs in virtual 100% of the cases refer patients with symptoms of schizophrenia or bipolar disorder to an in or out patients contact to a psychiatric hospital. Therefore, we include all persons with all levels of severity of the disorders.

In Denmark a major downsizing of psychiatric inpatient hospitalizations has occurred in the 1980's and 1990's where outpatient and community-based mental health services have been developed. From the late 1980's to 2007 the number of psychiatric beds have been halved [39]. To take this development into account we have included outpatient contacts from 1995. As we have no information of outpatient contacts before 1995 a slight under-representation of less severe cases of schizophrenia and bipolar disorder would occur in the start of the follow-up. However, patients in outpatient treatment tend to have many contacts, and the number of incorrectly assigned person-years would be limited.

Persons with severe mental disorder tend to live in the larger cities [40], with e.g. more pollution, which could have a potential effect on the excess mortality. However, the geographical variation in e.g. pollution and particular healthcare provision is rather small in Denmark; thus, we think that the potential effect of geographic variation must have a limited effect.

We have only included younger persons under the age of 52 in this study. This should be kept in mind in comparisons with other studies. We made this restriction because the Danish registries do not contain complete medical information on persons born before 1955. Moreover, excess mortality in persons with severe mental disorder is larger among younger persons [4], and thus particularly relevant from a clinical point of view with regard to prevention.

### Conclusion

In conclusion, mortality from natural causes is high in patients with schizophrenia or bipolar disorder compared with the general population, and we suggest that this excess mortality may partly be rooted in higher rates of somatic disorders and partly in under-detection or under-treatment of the chronic disorders.
